# Supplementary material for: Extended Infusion of Beta-Lactams and Glycopeptides: A New Era in Pediatric Care? A Systematic Review and Meta-Analysis
Source: Antibiotics (Basel). 2024 Feb 7;13(2):164. doi: 10.3390/antibiotics13020164 (PMC10886114; doi:10.3390/antibiotics13020164)
Supplement: Supplementary file 1 [file antibiotics-13-00164-s001.zip › antibiotics-2855936-supplementary.pdf]

antibiotics

MDPI

Supplementary Material

Supplement A: Differences from the Protocol and the Final Review

1. Outcome assessment: target attainment. Initially, we planned to assess the probability of target attainment only in prose, as quantifiable results were not expected. However, tor the glycopeptide subgroup, the pharmacokinetic targets were reported in a more detailed quantitative manner than anticipated. Consequently, we also evaluated this outcome using relative risk (RR) as measure of effect to determine statistical significance.

2. The third outcome was renamed from "microbiological cure" to "microbiological eradication" for improved clarity.

Supplement B: Search Keys & Strategies

Search keys used in Database

Databank	sign	Explanation
Pubmed	meshterm[Mesh]	Medical Subject heading, medical thesaurus for indexing.
	term*	Truncation, Endings can vary, but automatic indexing is lost
	“term”	Phrase search
	[pt]	Publication type filter
	“Term”[tiab]	Search of term in Title or abstract
	“term term”[tiab:~n]	Search of term in Title or abstract with a proximity of words of at least n
	#n	Search query n
EMBASE	term*	Truncation, Endings can vary, but automatic indexing is lost
	"term":	phrase
	/exp	Emtree term (major topic of the publication) exploded
	[randomized controlled trial]/lim	
	#n	Search query n
CENTRAL	term*	Truncation, Endings can vary, but automatic indexing is lost
	Mesh descriptor: [term]	
	“term”	Phrase search
	#n	Search query n

The concepts “population”, “study drug”, “intervention: mode of action”, and “outcome” were combined with the “AND” operator, (**Figure A1**). Search terms for each concept were determined using MeSH terms, free text, truncated words, and title & abstract filters. Pilot searches were conducted to estimate the number of retrieved studies.

The exact final search strategy for MEDLINE via PubMed is detailed below, while print-screens illustrate the exact search strategies for EMBASE and CENTRAL (Supplement C).

PubMed (Medline)
27.03.2023

**Population**	AND	**Study drug**	AND	**Intervention: mode of action**	AND	**Outcome**
"child"[Mesh] OR<br>"infant"[Mesh] OR<br>"adolescent"[Mesh] OR<br>child* OR<br>adolescent* OR<br>neonate* OR<br>pediatric* OR<br>paediatric* OR<br>infant* OR<br>child OR<br>children OR<br>adolescent OR<br>baby OR<br>babies OR<br>neonate OR<br>neonates OR<br>pediatric OR<br>paediatrics OR<br>paediatric OR<br>infant) NOT<br>adult[Mesh]		"beta-lactams"[Mesh] OR<br>"lipoglycopeptides"[Mesh] OR<br>"Vancomycin"[Mesh] OR<br>cephalosporin* OR<br>penicillin* OR<br>monobactam OR<br>carbapenem* OR<br>betalactam OR<br>β-lactam OR<br>β lactam OR<br>cephalosporin OR<br>ceftriaxone OR<br>cefotaxime OR<br>cefuroxime OR<br>cefepime OR<br>cefazolin OR<br>cefamandole OR<br>ceftobiprole OR<br>ceftaroline OR<br>penicillin OR<br>piperacillin OR<br>amoxicillin OR<br>ampicillin OR<br>flucloxacillin OR<br>benzylpenicillin OR<br>monobactam OR<br>aztreonam OR<br>carbapenem OR<br>meropenem OR<br>imipenem OR<br>ertapenem OR<br>glycopeptide OR<br>vancomycin OR<br>teicoplanin		continuous infusion* OR<br>continuous administration* OR<br>continuous application* OR<br>sustained infusion* OR<br>persistent infusion* OR<br>extended infusion* OR<br>extended application* OR<br>extended administration* OR<br>prolonged infusion* OR<br>prolonged administration* OR<br>prolonged application* OR<br>optimized dosing regimen* OR<br>continuous infusion OR<br>continuous administration OR<br>continuous dosing OR<br>continuous application OR<br>sustained infusion OR<br>persistent infusion OR<br>prolonged infusion OR<br>prolonged administration OR<br>prolonged application OR<br>optimized dosing regimen		"pharmacokinetics"[Mesh] OR<br>"pharmacokinetics" [Subheading] OR<br>pharmacokinetic* OR<br>blood culture* OR<br>pharmacokinetics OR<br>mortality OR<br>pk/pd OR<br>bacteriological cure OR<br>microbiological cure OR<br>blood culture OR<br>blood cultures OR<br>TDM OR<br>therapeutic drug monitoring OR<br>attainment target OR<br>attainment therapeutic drug<br>concentrations OR<br>clinical success OR<br>CRP OR<br>IL-6 OR<br>interleukin 6

Methodological changes (not predefined in the protocol)

Search: Due to limited results, we excluded the "comparison: mode of action" and "mode of administration: intravenous" concepts to enhance search sensitivity.

Supplement C: Search Strategies

1. Search with RCT filter

Pubmed Search with RCT filter: Date: 21.03.2023

History and Search Details

Search	Actions	Details	Query	Results	Time
#15	...		Search: **#1 AND #2 AND #3 AND #4 AND #14**	104	04:41:14
#16	...		Search: **34029707 OR 30700564 OR 31448789 OR 28980166 OR 27918382**	5	04:35:50
#14	...		Search: **#12 NOT #13**	1,403,651	04:31:02
#13	...		Search: **animals [mh] NOT humans [mh]**	5,102,198	04:30:51
#12	...		Search: **#5 OR #6 OR #7 OR #8 OR #9 OR #10 OR #11**	1,524,435	04:30:35
#11	...		Search: **trial [ti]**	281,324	04:30:18
#10	...		Search: **randomly [tiab]**	405,179	04:29:56
#9	...		Search: **clinical trials as topic [mesh:noexp]**	200,890	04:29:44
#8	...		Search: **placebo [tiab]**	243,458	04:29:34
#7	...		Search: **randomized [tiab]**	650,797	04:29:24
#6	...		Search: **controlled clinical trial [pt]**	680,402	04:29:14
#5	...		Search: **randomized controlled trial [pt]**	590,088	04:27:42
#4	...		Search: **("Pharmacokinetics"[Mesh]) OR ("pharmacokinetics"[Subheading]) OR (pharmacokinetic*) OR (blood culture*) OR (pharmacokinetics) OR (mortality) OR (pk/pd) OR (bacteriological cure) OR (microbiological cure) OR (blood culture) OR (blood cultures) OR (TDM) OR (therapeutic drug monitoring) OR (attainment target) OR (attainment therapeutic drug concentrations) OR (attainment therapeutic concentrations) OR (clinical success) OR (CRP) OR (IL-6) OR (interleukin 6)**	3,051,841	04:27:32
#3	...		Search: **(continuous infusion*) OR (continuous administration*) OR (continuous application*) OR (sustained infusion*) OR (persistent infusion*) OR (extended infusion*) OR (extended application*) OR (extended administration*) OR (prolonged infusion*) OR (prolonged administration*) OR (prolonged application*) OR (optimized dosing regimen*) OR (continuous infusion) OR (continuous administration) OR (continuous dosing) OR (continuous application) OR (sustained infusion) OR (persistent infusion) OR (extended dosing) OR (extended infusion) OR (extended application) OR (extended administration) OR (prolonged infusion) OR (prolonged administration) OR (prolonged application) OR (optimized dosing regimen)**	613,298	04:27:17
#2	...		Search: **("beta-Lactams"[Mesh]) OR ("Lipoglycopeptides"[Mesh]) OR ("Vancomycin"[Mesh]) OR (cephalosporin*) OR (penicillin*) OR (monobactam*) OR (carbapenem*) OR (glycopeptide*) OR (beta lactam) OR (beta-lactam) OR (betalactam) OR (β-lactam) OR (β lactam) OR (cephalosporin) OR (ceftriaxone) OR (cefotaxime) OR (cefuroxime) OR (cefepime) OR (cefazolin) OR (cefamandole) OR (ceftobiprole) OR (ceftaroline) OR (penicillin) OR (piperacillin) OR (amoxicillin) OR (ampicillin) OR (flucloxacillin) OR (benzylpenicillin) OR (monobactam) OR (aztreonam) OR (carbapenem) OR (meropenem) OR (imipenem) OR (Ertapenem) OR (glycopeptide) OR (vancomycin) OR (teicoplanin)**	297,358	04:26:31
#1	...		Search: **(("Child"[Mesh]) OR ("Infant"[Mesh]) OR ("Adolescent"[Mesh]) OR (child*) OR (adolescent*) OR (neonate*) OR (pediatric*) OR (paediatric*) OR(infant*) OR (child) OR (children) OR (adolescent) OR (baby) OR (babies) OR (neonate) OR (neonates) OR (pediatric) OR (paediatrics) OR (paediatric) OR (infant)) NOT "Adult"[Mesh]**	3,185,794	04:26:15

Embase Search With RCT filter: Date: 21.03.2023

No.	Query	Results
#42	#40 AND #41	4
#41	34029707 OR 30700564 OR 31448789 OR 28980166 OR 27918382	6
#40	#1 AND #2 AND #3 AND #4 AND #39	331
#39	#24 NOT #38	5,442,959
#38	#25 OR #26 OR #27 OR #28 OR #29 OR #30 OR #31 OR #32 OR #33 OR #34 OR #35 OR #36 OR #37	4,215,332
#37	'animal experiment'/de NOT ('human experiment'/de OR 'human'/de)	2,515,415
#36	(rat:ti,tt OR rats:ti,tt OR mouse:ti,tt OR mice:ti,tt OR swine:ti,tt OR porcine:ti,tt OR murine:ti,tt OR sheep:ti,tt OR lambs:ti,tt OR pigs:ti,tt OR piglets:ti,tt OR rabbit:ti,tt OR rabbits:ti,tt OR cat:ti,tt OR cats:ti,tt OR dog:ti,tt OR dogs:ti,tt OR cattle:ti,tt OR bovine:ti,tt OR monkey:ti,tt OR monkeys:ti,tt OR trout:ti,tt OR marmoset*:ti,tt) AND 'animal experiment'/de	1,197,913
#35	(databases NEAR/5 searched):ab	62,052
#34	'update review':ab	134
#33	'we searched':ab AND (review:ti,tt OR review:it)	46,502
#32	review:ab AND review:it NOT trial:ti,tt	1,065,824
#31	('random cluster' NEAR/4 sampl*):ti,ab,tt	1,635
#30	'random field*':ti,ab,tt	2,833
#29	nonrandom*:ti,ab,tt NOT random*:ti,ab,tt	18,493
#28	'systematic review':ti,tt NOT (trial:ti,tt OR study:ti,tt)	239,923
#27	'case control*':ti,ab,tt AND random*:ti,ab,tt NOT ('randomised controlled':ti,ab,tt OR 'randomized controlled':ti,ab,tt)	20,796
#26	'cross-sectional study' NOT ('randomized controlled trial'/de OR 'controlled clinical study'/de OR 'controlled study'/de OR 'randomised controlled':ti,ab,tt OR 'randomized controlled':ti,ab,tt OR 'control group':ti,ab,tt OR 'control groups':ti,ab,tt)	363,762
#25	((random* NEXT/1 sampl* NEAR/8 ('cross section*' OR questionnaire* OR survey OR surveys OR database OR databases)):ti,ab,tt) NOT ('comparative study'/de OR 'controlled study'/de OR 'randomised controlled':ti,ab,tt OR 'randomized controlled':ti,ab,tt OR 'randomly assigned':ti,ab,tt)	3,032
#24	#5 OR #6 OR #7 OR #8 OR #9 OR #10 OR #11 OR #12 OR #13 OR #14 OR #15 OR #16 OR #17 OR #18 OR #19 OR #20 OR #21 OR #22 OR #23	6,156,764
#23	trial:ti,tt	391,234
#22	'human experiment'/de	619,001
#21	volunteer:ti,ab,tt OR volunteers:ti,ab,tt	278,578
#20	(controlled NEAR/8 (study OR design OR trial)):ti,ab,tt	442,908
#19	assigned:ti,ab,tt OR allocated:ti,ab,tt	473,638
#18	((assign* OR match OR matched OR allocation) NEAR/6 (alternate OR group OR groups OR intervention OR interventions OR patient OR patients OR subject OR subjects OR participant OR participants)):ti,ab,tt	442,621
#17	crossover:ti,ab,tt OR 'cross over':ti,ab,tt	121,608
#16	(parallel NEXT/1 group*):ti,ab,tt	31,073
#15	'double blind procedure'/de	205,178
#14	((double OR single OR doubly OR singly) NEXT/1 (blind OR blinded OR blindly)):ti,ab,tt	269,176
#13	(open NEXT/1 label):ti,ab,tt	104,334
#12	(evaluated:ab OR evaluate:ab OR evaluating:ab OR assessed:ab OR assess:ab) AND (compare:ab OR compared:ab OR comparing:ab OR comparison:ab)	2,667,976
#11	compare:ti,tt OR compared:ti,tt OR comparison:ti,tt	612,801
#10	placebo:ti,ab,tt	356,732
#9	'intermethod comparison'/de	295,914
#8	'randomization'/de	96,452
#7	random*:ti,ab,tt	1,901,461
#6	'controlled clinical trial'/de	439,420
#5	'randomized controlled trial'/de	756,139
#4	'pharmacokinetics'/exp OR pharmacokinetic* OR (blood AND culture*) OR pharmacokinetics OR mortality OR pk/pd OR (bacteriological NEAR/3 cure) OR (microbiological NEAR/3 cure) OR (blood NEAR/3 culture) OR (blood NEAR/3 cultures) OR tdm OR (therapeutic NEAR/3 drug NEAR/3 monitoring) OR (attainment NEAR/3 target) OR (attainment NEAR/3 therapeutic NEAR/3 concentrations) OR (clinical NEAR/3 success) OR crp OR 'il 6'	3,504,367
#3	'continuous infusion'/exp OR ((continuous* OR sustain* OR persistant* OR extended* OR prolong* OR consistent) NEAR/3 (infusion* OR administrat* OR applicati* OR dosing*)) OR (continuous AND infusion*) OR (continuous AND administration*) OR (continuous AND application*) OR (sustained AND infusion*) OR (persistent AND infusion*) OR (extended AND infusion*) OR (extended AND application*) OR (extended AND administration*) OR (prolonged AND infusion*) OR (prolonged AND administration*) OR (prolonged AND application*) OR (optimized AND dosing AND regimen*) OR (continuous AND infusion) OR (continuous AND administration) OR (continuous AND dosing) OR (continuous AND application) OR (sustained AND infusion) OR (persistent AND infusion) OR (extended AND dosing) OR (extended AND infusion) OR (extended AND application) OR (extended AND administration) OR (prolonged AND infusion) OR (prolonged AND administration) OR (prolonged AND application) OR (optimized AND dosing AND regimen)	420,076
#2	'beta lactam antibiotic'/exp OR 'beta lactam'/exp OR 'glycopeptide'/exp OR cephalosporin* OR penicillin* OR (beta AND lactam) OR 'beta lactam' OR betalactam OR 'β lactam' OR (β AND lactam) OR cephalosporin OR ceftriaxone OR cefotaxime OR cefuroxime OR cefepime OR cefazolin OR cefamandole OR ceftobiprole OR ceftaroline OR penicillin OR piperacillin OR amoxicillin OR ampicillin OR flucloxacillin OR benzylpenicillin OR monobactam OR aztreonam OR carbapenem OR meropenem OR imipenem OR ertapenem OR glycopeptide OR vancomycin OR teicoplanin	680,890
#1	'child' OR 'infant' OR 'newborn'/exp OR 'newborn' OR 'pediatric' OR 'adolescent' OR child* OR 'youth'/exp OR youth OR adolescent* OR neonate* OR pediatric* OR paediatric* OR infant* OR 'child'/exp OR child OR 'children'/exp OR children OR 'adolescent'/exp OR adolescent OR 'neonate'/exp OR neonate OR neonates OR 'pediatric'/exp OR pediatric OR 'pediatrics'/exp OR pediatrics OR 'paediatric'/exp OR paediatric OR 'paediatrics'/exp OR paediatrics OR 'infant'/exp OR infant OR 'baby'/exp OR baby OR babies	6,194,127

Cochrane Search with RCT filter: Date: 21.03.2023

Search Name: cochrane_search_2023_03_21
Date Run: 22.03.2023 15:00

ID	Search	Hits
#1	child* OR adolescent* OR neonate* OR pediatric* OR paediatric* OR infant* OR child OR children OR adolescent OR babies OR neonate OR neonates OR pediatric OR pediatrics OR paediatric OR paediatrics OR infant OR baby OR babies OR youth OR teenage OR teenager	349458
#2	MeSH descriptor: [beta-Lactams] explode all trees	10440
#3	cephalosporin* OR penicillin* OR (beta AND lactam) OR 'beta lactam' OR betalactam OR 'β lactam' OR (β AND lactam) OR cephalosporin OR ceftriaxone OR cefotaxime OR cefuroxime OR cefepime OR cefazolin OR cefamandole OR ceftobiprole OR ceftaroline OR penicillin OR piperacillin OR amoxicillin OR ampicillin OR flucloxacillin OR benzylpenicillin OR monobactam OR aztreonam OR carbapenem OR meropenem OR imipenem OR ertapenem OR glycopeptide OR vancomycin OR teicoplanin	20414
#4	#2 OR #3	21519
#5	(continuous AND infusion*) OR (continuous AND administration*) OR (continuous AND application*) OR (sustained AND infusion*) OR (persistent AND infusion*) OR (extended AND infusion*) OR (extended AND application*) OR (extended AND administration*) OR (prolonged AND infusion*) OR (prolonged AND administration*) OR (prolonged AND application*) OR (optimized AND dosing AND regimen*) OR (continuous AND infusion) OR (continuous AND administration) OR (continuous AND dosing) OR (continuous AND application) OR (sustained AND infusion) OR (persistent AND infusion) OR (extended AND dosing) OR (extended AND infusion) OR (extended AND application) OR (extended AND administration) OR (prolonged AND infusion) OR (prolonged AND administration) OR (prolonged AND application) OR (optimized AND dosing AND regimen)	56130
#6	MeSH descriptor: [Pharmacokinetics] explode all trees	17455
#7	'pharmacokinetic* OR (blood AND culture*) OR pharmacokinetics OR mortality OR "pk/pd" OR (bacteriological AND cure) OR (microbiological AND cure) OR (blood AND culture) OR (blood AND cultures) OR tdm OR (therapeutic AND drug AND monitoring) OR (attainment AND of AND target) OR (attainment AND of AND therapeutic AND drug AND concentrations) OR (attainment AND of AND therapeutic AND concentrations) OR (clinical AND success) OR crp OR "il 6" OR (interleukin AND 6)	284280
#8	#6 OR #7	288865
#9	#1 AND #4 AND #5 #8	492
Filter	Trials	146

2. Search strategies including observational studies:
Pubmed final search including observational studies: Date: 23.03.2023

History and Search Details

Search	Actions	Details	Query	Results	Time
#5	...		Search: **#1 AND #2 AND #3 AND #4**	774	08:47:23
#4	...		Search: **("Pharmacokinetics"[Mesh]) OR ("pharmacokinetics"[Subheading]) OR (pharmacokinetic*) OR (blood culture*) OR (pharmacokinetics) OR (mortality) OR (pk/pd) OR (bacteriological cure) OR (microbiological cure) OR (blood culture) OR (blood cultures) OR (TDM) OR (therapeutic drug monitoring) OR (attainment target) OR (attainment therapeutic drug concentrations) OR (attainment therapeutic concentrations) OR (clinical success) OR (CRP) OR (IL-6) OR (interleukin 6)**	3,054,092	08:47:05
#3	...		Search: **(continuous infusion*) OR (continuous administration*) OR (continuous application*) OR (sustained infusion*) OR (persistent infusion*) OR (extended infusion*) OR (extended application*) OR (extended administration*) OR (prolonged infusion*) OR (prolonged administration*) OR (prolonged application*) OR (optimized dosing regimen*) OR (continuous infusion) OR (continuous administration) OR (continuous dosing) OR (continuous application) OR (sustained infusion) OR (persistent infusion) OR (extended dosing) OR (extended infusion) OR (extended application) OR (extended administration) OR (prolonged infusion) OR (prolonged administration) OR (prolonged application) OR (optimized dosing regimen)**	613,731	08:46:50
#2	...		Search: **("beta-Lactams"[Mesh]) OR ("Lipoglycopeptides"[Mesh]) OR ("Vancomycin"[Mesh]) OR (cephalosporin*) OR (penicillin*) OR (monobactam*) OR (carbapenem*) OR (glycopeptide*) OR (beta lactam) OR (beta-lactam) OR (betalactam) OR (β-lactam) OR (β lactam) OR (cephalosporin) OR (ceftriaxone) OR (cefotaxime) OR (cefuroxime) OR (cefepime) OR (cefazolin) OR (cefamandole) OR (ceftobiprole) OR (ceftaroline) OR (penicillin) OR (piperacillin) OR (amoxicillin) OR (ampicillin) OR (flucloxacillin) OR (benzylpenicillin) OR (monobactam) OR (aztreonam) OR (carbapenem) OR (meropenem) OR (imipenem) OR (Ertapenem) OR (glycopeptide) OR (vancomycin) OR (teicoplanin)**	297,551	08:46:39
#1	...		Search: **(("Child"[Mesh]) OR ("Infant"[Mesh]) OR ("Adolescent"[Mesh]) OR (child*) OR (adolescent*) OR (neonate*) OR (pediatric*) OR (paediatric*) OR(infant*) OR (child) OR (children) OR (adolescent) OR (baby) OR (babies) OR (neonate) OR (neonates) OR (pediatric) OR (paediatrics) OR (paediatric) OR (infant)) NOT "Adult"[Mesh]**	3,188,917	08:46:20

Embase final search including observational studies: Date: 27.03.2023

:023 Exported Print HTML | Embase

Embase®

Embase Session Results

No.	Query	Results
#5	#1 AND #2 AND #3 AND #4	2,146
#4	'pharmacokinetics'/exp OR pharmacokinetic* OR (blood AND culture*) OR pharmacokinetics OR mortality OR pk/pd OR (bacteriological NEAR/3 cure) OR (microbiological NEAR/3 cure) OR (blood NEAR/3 culture) OR (blood NEAR/3 cultures) OR tdm OR (therapeutic NEAR/3 drug NEAR/3 monitoring) OR (attainment NEAR/3 target) OR (attainment NEAR/3 therapeutic NEAR/3 concentrations) OR (clinical NEAR/3 success) OR crp OR 'il 6'	3,507,720
#3	'continuous infusion'/exp OR ((continuous* OR sustain* OR persistant* OR extended* OR prolong* OR consistent) NEAR/3 (infusion* OR administrat* OR applicati* OR dosing*)) OR (continuous AND infusion*) OR (continuous AND administration*) OR (continuous AND application*) OR (sustained AND infusion*) OR (persistent AND infusion*) OR (extended AND infusion*) OR (extended AND application*) OR (extended AND administration*) OR (prolonged AND infusion*) OR (prolonged AND administration*) OR (prolonged AND application*) OR (optimized AND dosing AND regimen*) OR (continuous AND infusion) OR (continuous AND administration) OR (continuous AND dosing) OR (continuous AND application) OR (sustained AND infusion) OR (persistent AND infusion) OR (extended AND dosing) OR (extended AND infusion) OR (extended AND application) OR (extended AND administration) OR (prolonged AND infusion) OR (prolonged AND administration) OR (prolonged AND application) OR (optimized AND dosing AND regimen)	420,487
#2	'beta lactam antibiotic'/exp OR 'beta lactam'/exp OR 'glycopeptide'/exp OR cephalosporin* OR penicillin* OR (beta AND lactam) OR 'beta lactam' OR betalactam OR 'β lactam' OR (β AND lactam) OR cephalosporin OR ceftriaxone OR cefotaxime OR cefuroxime OR cefepime OR cefazolin OR cefamandole OR ceftobiprole OR ceftaroline OR penicillin OR piperacillin OR amoxicillin OR ampicillin OR flucloxacillin OR benzylpenicillin OR monobactam OR aztreonam OR carbapenem OR meropenem OR imipenem OR ertapenem OR glycopeptide OR vancomycin OR teicoplanin	681,502
#1	'newborn'/exp OR 'newborn' OR child* OR 'youth' OR 'youth'/exp OR youth OR adolescent* OR neonate* OR pediatric* OR paediatric* OR infant* OR 'child' OR 'child'/exp OR child OR 'children' OR 'children'/exp OR children OR 'adolescent' OR 'adolescent'/exp OR adolescent OR 'neonate' OR 'neonate'/exp OR neonate OR neonates OR 'pediatric' OR 'pediatric'/exp OR pediatric OR 'pediatrics' OR 'pediatrics'/exp OR pediatrics OR 'paediatric' OR 'paediatric'/exp OR paediatric OR 'paediatrics' OR 'paediatrics'/exp OR paediatrics OR 'infant' OR 'infant'/exp OR infant OR 'baby' OR 'baby'/exp OR baby OR babies	6,199,233

Cochrane final search: Date: 21.03.2023

Search Name: cochrane_search_2023_03_21
Date Run: 22.03.2023 15:00

ID	Search	Hits
#1	child* OR adolescent* OR neonate* OR pediatric* OR paediatric* OR infant* OR child OR children OR adolescent OR babies OR neonate OR neonates OR pediatric OR pediatrics OR paediatric OR paediatrics OR infant OR baby OR babies OR youth OR teenage OR teenager	349458
#2	MeSH descriptor: [beta-Lactams] explode all trees	10440
#3	cephalosporin* OR penicillin* OR (beta AND lactam) OR 'beta lactam' OR betalactam OR 'β lactam' OR (β AND lactam) OR cephalosporin OR ceftriaxone OR cefotaxime OR cefuroxime OR cefepime OR cefazolin OR cefamandole OR ceftobiprole OR ceftaroline OR penicillin OR piperacillin OR amoxicillin OR ampicillin OR flucloxacillin OR benzylpenicillin OR monobactam OR aztreonam OR carbapenem OR meropenem OR imipenem OR ertapenem OR glycopeptide OR vancomycin OR teicoplanin	20414
#4	#2 OR #3	21519
#5	(continuous AND infusion*) OR (continuous AND administration*) OR (continuous AND application*) OR (sustained AND infusion*) OR (persistent AND infusion*) OR (extended AND infusion*) OR (extended AND application*) OR (extended AND administration*) OR (prolonged AND infusion*) OR (prolonged AND administration*) OR (prolonged AND application*) OR (optimized AND dosing AND regimen*) OR (continuous AND infusion) OR (continuous AND administration) OR (continuous AND dosing) OR (continuous AND application) OR (sustained AND infusion) OR (persistent AND infusion) OR (extended AND dosing) OR (extended AND infusion) OR (extended AND application) OR (extended AND administration) OR (prolonged AND infusion) OR (prolonged AND administration) OR (prolonged AND application) OR (optimized AND dosing AND regimen)	56130
#6	MeSH descriptor: [Pharmacokinetics] explode all trees	17455
#7	'pharmacokinetic* OR (blood AND culture*) OR pharmacokinetics OR mortality OR "pk/pd" OR (bacteriological AND cure) OR (microbiological AND cure) OR (blood AND culture) OR (blood AND cultures) OR tdm OR (therapeutic AND drug AND monitoring) OR (attainment AND of AND target) OR (attainment AND of AND therapeutic AND drug AND concentrations) OR (attainment AND of AND therapeutic AND concentrations) OR (clinical AND success) OR crp OR "il 6" OR (interleukin AND 6)	284280
#8	#6 OR #7	288865
#9	#1 AND #4 AND #5 #8	492
Filter	Trials	146

Supplement D: Calculation of Variables

Table S1 was created to facilitate calculation. The RR (equation 3) was calculated by dividing the risk of the intervention group (COI) (equation 1) by the risk of the comparison group (IA) (equation 2).

Table S1: Intervention and Outcome Table used for relative risk calculation.

	Outcome observed	Outcome not observed	total
Continuous infusion / prolonged Infusion	a	b	a + b
Intermittent administration	c	d	c + d

$$Risk\ COI\ =\ \frac{a}{a+b} \tag{1}$$

$$Risk\ IA\ =\ \frac{c}{c+d} \tag{2}$$

$$Relative\ Risk\ (RR)\ =\ \frac{Risk\ CI}{Risk\ IA}\ =\ \frac{a}{a+b}/\frac{c}{c+d} \tag{3}$$

The standard error (SE) was calculated (equation 4) as an intermediate step to be able to calculate the 95% confidence interval (CI) for each outcome (equation 5).

P- Values: P-values were calculated in R according to Altman et al. [1] and reported adhering to the guideline from Aguinis et al [2].

$$Standard\ error\ (SE)\ =\ \sqrt{\frac{1}{a}+\frac{1}{c}-\frac{1}{a+b}-\frac{1}{c+d}} \tag{4}$$

$$95\%\ Confidence\ Interval\ (CI)\ =\ e^{ln(RR)\ \pm\ 1.96*SE} \tag{5}$$

The inverse variance was calculated via standard error (SE), since for relative Risks, the standard deviation (SD) is equivalent to the SE (equation 6 & 7).

$$Var\ =\ SD^2\ =\ SE^2 \tag{6}$$

$$Study\ weight\ =\ \frac{1}{Var}\ =\ \frac{1}{SE^2} \tag{7}$$

Supplement E: Reason for Exclusion of Articles in Full Text Review

Table S2. : Reason for Exclusion of Articles in Full Text Review

Author & Year	Title	Reason for exclusion
Berthaud, 2019	Early Bayesian Dose Adjustment of Vancomycin Continuous Infusion in Children: a Randomized Controlled Trial	No comparison of COI vs. IA
Cies, 2017	Population Pharmacokinetics and Pharmacodynamic Target Attainment of Meropenem in Critically Ill Young Children	In Silico models, no observational design or RCT, no comparison of COI vs IA
Debray, 2023	Beta-lactam exposure and safety in intermittent or continuous infusion in critically ill children: an observational monocentric study	CRRT included: almost 1/3 of all samples

Imburgia, 2022	Evaluation of the safety of cefepime prolonged infusions in paediatric patients with cystic fibrosis	Participants with antibiotic allergy included
Jaruratanasirikul, 2010	Comparison of continuous infusion versus intermittent infusion of vancomycin in patients with methicillin-resistant Staphylococcus aureus	Adults included
Jaruratanasirikul, 2003	Comparison of the pharmacodynamics of meropenem in healthy volunteers following administration by intermittent infusion or bolus injection	Adults included
Wang, 2022	Improving the efficacy for meropenem therapy requires a high probability of target attainment in critically ill infants and children	No comparison of COI vs. IA
Wu, 2022	Clinical utility of a model-based piperacillin dose in neonates with early-onset sepsis	No comparison of COI vs. IA

Supplement F: Risk of Bias (ROB) Judgement

ROB judgment

Study ID	Experimental	Comparator	Outcome	D1	D2	D3	D4	D5	Overall
Solorzano 2019	CI	IA	1 Mortality	!	+	+	+	-	-
Solorzano 2019	CI	IA	2 Clinical success	-	!	+	!	!	-
Chongcharoenyanon 2021	PI	IA	1 Mortality	+	+	+	+	!	!
Chongcharoenyanon 2021	PI	IA	2 Clinical success	+	+	+	+	-	-
Chongcharoenyanon 2021	PI	IA	4 Target attainment	+	+	+	+	!	!
Shabaan 2017	PI	IA	1 Mortality	+	+	+	+	+	+
Shabaan 2017	PI	IA	2 Clinical success	+	+	+	+	+	+
Shabaan 2017	PI	IA	3 Bacteriological Cure	+	+	+	+	+	+
Gwee 2019	CI	IA	4 Target attainment	+	!	+	+	+	!
Gwee 2019	CI	IA	1 Mortality	+	+	+	+	+	+

Legend

+	Low risk
!	Some concerns
-	High risk
D1	Randomisation process
D2	Deviations from the intended interventions
D3	Missing outcome data
D4	Measurement of the outcome
D5	Selection of the reported result

ROBINS judgment

Study: Beauchamp 2019

Domain	Outcome 1: Mortality	Outcome 2: Clinical success	Outcome 3: Microbiological eradication
Bias due to confounding	Serious risk	Serious Risk	Serious risk
Bias in selection of participants	Serious risk	Serious Risk	Serious risk
Bias in classification of interventions	Moderate risk	Moderate risk	Moderate risk
Bias due to deviations from intended interventions	Serious risk	Serious risk	Serious risk
Bias due to missing data	Low risk	Low risk	Low risk
Bias in measurement of outcomes	Low risk	Moderate risk	Low risk
Bias in selection of the reported result	Low risk	Low risk	Low risk
**Overall**	**Serious risk**	**Serious risk**	**Serious risk**

(outcome 4 Attainment of target concentration : n/a)

Study: Zembles 2021

Domain	Outcome 1: Mortality	Outcome 2: Clinical success	Outcome 3: Microbiological eradication
Bias due to confounding	Low risk	Low risk	n/a
Bias in selection of participants	Low risk	Low risk	n/a
Bias in classification of interventions	Low risk	Low risk	n/a
Bias due to deviations from intended interventions	Low risk	Low risk	n/a
Bias due to missing data	Low risk	Low risk	n/a
Bias in measurement of outcomes	Moderate risk	Moderate risk	n/a
Bias in selection of the reported result	Moderate risk	Moderate risk	n/a
**Overall**	**Moderate risk**	**Moderate risk**	**n/a**

(outcome 4 Attainment of target concentration : n/a)

Study: Zembles 2022

Domain	Outcome 1: Mortality	Outcome 2: Clinical success	Outcome 3: Microbiological eradication
Bias due to confounding	Moderate risk	Moderate risk	n/a
Bias in selection of participants	Low risk	Low risk	n/a
Bias in classification of interventions	Low risk	Low risk	n/a
Bias due to deviations from intended interventions	Low risk	Low risk	n/a
Bias due to missing data	Low risk	Low risk	n/a
Bias in measurement of outcomes	Moderate risk	Moderate risk	n/a
Bias in selection of the reported result	Low risk	Low risk	n/a
**Overall**	**Moderate risk**	**Moderate risk**	**n/a**

(outcome 4 Attainment of target concentration : n/a)

Study: Demirel 2015

Domain	Outcome 1: Mortality	Outcome 2: Clinical success	Outcome 3: Microbiological eradication	Outcome 4: PTA
Bias due to confounding	Serious risk	Serious risk	Serious risk	Serious risk
Bias in selection of participants	Low risk	Low risk	Low risk	Low risk
Bias in classification of interventions	Low risk	Low risk	Low risk	Low risk
Bias due to deviations from intended interventions	Moderate risk	Moderate risk	Moderate risk	Moderate risk
Bias due to missing data	Low risk	Low risk	Serious risk	Low Risk
Bias in measurement of outcomes	Moderate risk	Moderate risk	Moderate risk	Moderate Risk
Bias in selection of the reported result	Low risk	Low risk	Low risk	Low risk
**Overall**	**Serious risk**	**Serious risk**	**Serious risk**	**Serious risk**

Study: Wysocki 2022

**Domain**	**Outcome 1: Mortality**	**Outcome 2: Clinical success**	**Outcome 3: Microbiological eradication**	**Outcome 4 PTA**
Bias due to confounding	Serious risk	Serious risk	Serious risk	Moderate risk
Bias in selection of participants	Low risk	Low risk	Low risk	Low risk
Bias in classification of interventions	Low risk	Low risk	Low risk	Low risk
Bias due to deviations from intended interventions	Low risk	Low risk	Low risk	Low risk
Bias due to missing data	Low risk	Low risk	Low risk	Low risk
Bias in measurement of outcomes	Serious risk	Serious risk	Serious risk	Serious risk
Bias in selection of the reported result	Low risk	Low risk	Low risk	Low risk
**Overall**	**Serious risk**	**Serious risk**	**Serious risk**	**Serious risk**

Exclusion of Wysocky study:

Immortal-time bias arose form the analysis of patients consistently receiving IA first and the then COI. This introduced bias to all outcomes including mortality, clinical success, microbiological eradication. Consequently, the study was excluded from the analysis. However, outcome analysis was unattainable as no deaths were reported, and there was no data available for clinical success or microbiological eradication.

Supplement G: Sensitivity Analyses

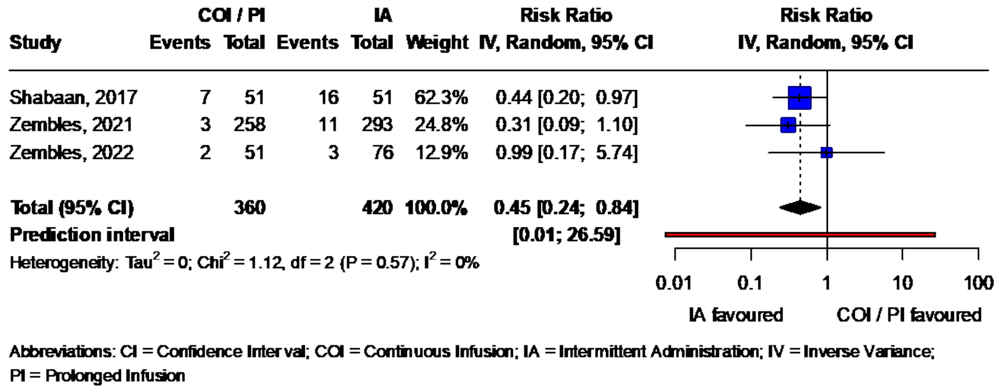

Figure S1. Outcome mortality without "high risk of bias" studies.

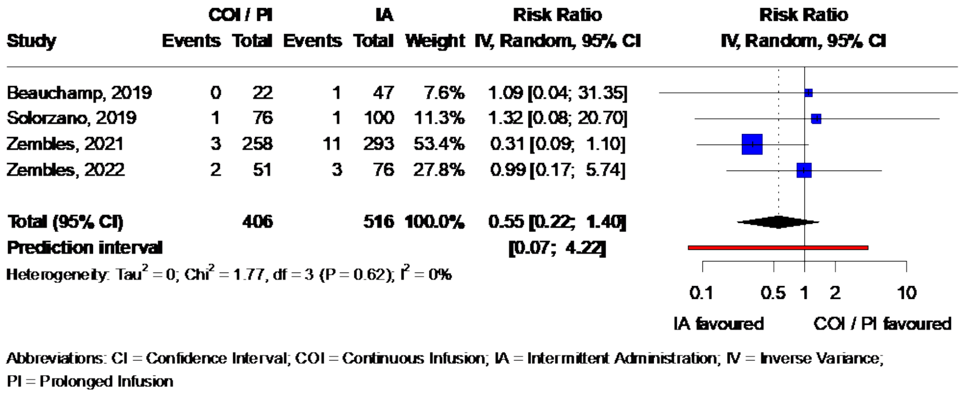

Figure S2. Outcome mortality without Shabaan, 2017. (only study with a P-value of < 0.05).

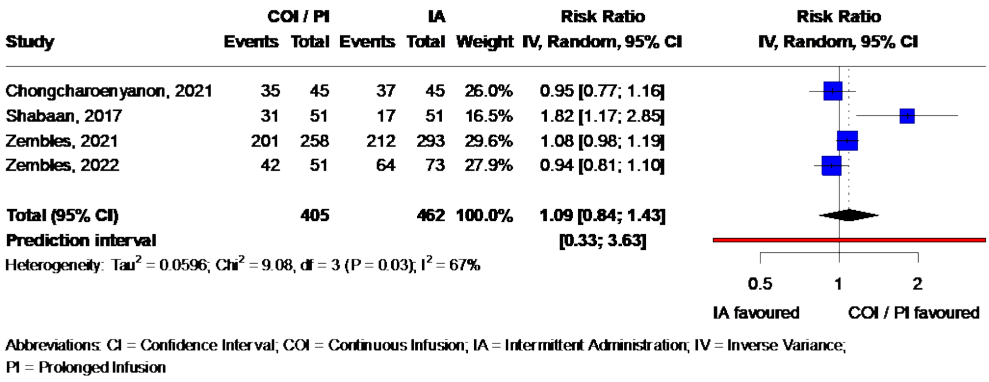

Figure S3. Outcome clinical success without "high risk of bias" studies.

Supplement H: Summary of Findings Tables for all Outcomes

Beta-lactam studies comprised three RCTs [3-5] and three observational studies [6-8]. The antibiotics investigated were piperacillin/tazobactam (PZT) [4,5], cefepime (FEP) [8], meropenem (MEM) [3] and a combination of FEP, MEM and PZT [6,7]. One beta-lactam study explored COI as an intervention [4], while the others examined PI lasting three to four hours. The total participant count was 1110 with an age range of 0 – 17 years, including one study exclusively involving neonates [3]. For glycopeptides, all studies investigated COI for vancomycin, enrolling a total of 209 patients. Two studies focused solely on newborns (< 34 weeks gestational age / 90 days of life) [9,10], while the third study included patients from 0 – 17 years [11]. **Tables S3 – S5** summarize the findings for each primary outcome.

Table S3. Summary of findings table for the outcome “mortality”.

Study	Antibiotic	Duration / Interval		Results Outcome Mortality		
		**IA,**	**PI & COI**	**Definition of outcome**	**RR / 95% CI**	**Significance: (RR = 1 not in 95% CI) / P-value**
Beauchamp, 2019	FEP	30 min Infusion / every 8 h	4 h infusion / every 8 h	Infection related mortality within 14 days of antibiotic therapy start.	1.09 / 0.04 – 31.35	No / 0.96
Chongcharoenyanon, 2021	TZP	30 min Infusion / every 8 h	4 h infusion / every 8 h	Not specified	n/a, absolute mortality 0 in both groups / n/a	n/a
Shabaan, 2017	MEM	30 min Infusion / every 8 h	4 h infusion / every 8 h	Not specified	0.44 / 0.20 – 0.97	Yes / 0.04
Solorzano, 2019	TZP	30 min Infusion / every 8 h	24 h infusion / n/a	Death during hospital stay	1.32 / 0.08 – 20.70	No / 0.85
Zembles, 2021	FEP, MEM, TZP	Infusion 15 - 30 min / varying	FEP & TZP: 4 h infusion / every 8 h; MEM: 3 h infusion / every 8 h;	All-cause mortality within 30 days of antibiotic completion	0.31 / 0.09 – 1.10	No / 0.07
Zembles, 2022	FEP, MEM, TZP	< 30 min Infusion / varying	3 - 4 h Infusion / every 8 hours	All-cause mortality within 30 days	0.99 / 0.17 – 5.74	No / 0.99
Demirel, 2015	VAN	Infusion 60 min / varying	24 h infusion / n/a	Death due to vancomycin sensitive enterococci	n/a (c = 0) / n/a	n/a
Gwee, 2019	VAN	Infusion 60 min / varying	24 h infusion / n/a	Death because of sepsis	n/a (a & c = 0) / n/a	n/a
Wysocki, 2022	VAN	n/a / varying	COI, n/a / n/a	All-cause mortality within 30 days	n/a (a & c = 0) / n/a	n/a

Abbreviations: AB = Antibiotic; CI = Confidence Interval; COI = Continuous Infusion; FEP = Cefepime; h = hour; IA: Intermittent Administration; MEM = Meropenem; min = minutes; PI = Prolonged Infusion; RR = Relative Risk; TZP = Piperacillin & Tazobactam; VAN = Vancomycin

Table S4. Summary of findings table for the outcome "clinical success".

Study	Antibiotic	Duration / Interval		Results Outcome Clinical Success		
		**IA,**	**PI & COI**	**Definition of outcome**	**RR / 95% CI**	**Significance: (RR = 1 not in 95% CI) / P-value**
Beauchamp, 2019	FEP	30 min Infusion / every 8 h	4 h infusion / every 8 h	Absence of Treatment failure	0.97 / 0.88 – 1.08	No / 0.61
Chongcharoenyanon, 2021	TZP	30 min Infusion / every 8 h	4 h infusion / every 8 h	Discharged after 30 days	0.95 / 0.77 – 1.16	No / 0.60
Shabaan, 2017	MEM	30 min Infusion / every 8 h	4 h infusion / every 8 h	Complete resolution of clinical signs and symptoms of sepsis at the end of therapy (hemodynamic stability, normal ABG values, temperature stability, tolerance for enteral feeding and discontinuation of inotropes for at least 48-hour duration)	1.82 / 1.17 – 2.85	Yes / 0.008
Solorzano, 2019	TZP	30 min Infusion / every 8 h	24 h infusion / n/a	Absence of treatment failure	1.01 / 0.88 – 1.16	No / 0.88
Zembles, 2021	FEP, MEM, TZP	Infusion 15 - 30 min / varying	FEP & TZP: 4 h infusion / every 8 h; MEM: 3 h infusion / every 8 h;	No readmission within first 30 days	1.08 / 0.98 – 1.19	No / 0.13
Zembles, 2022	FEP, MEM, TZP	< 30 min Infusion / varying	3 - 4 h Infusion / every 8 hours	No readmission within first 30 days	0.94 / 0.81 – 1.10	No / 0.42
Demirel, 2015	VAN	Infusion 60 min / varying	24 h infusion / n/a	No clinical failiure	0.94 / 0.87 – 1.02	No / 0.22
Gwee, 2019	VAN	Infusion 60 min / varying	24 h infusion / n/a	n/a	n/a / n/a	n/a

Wysocki, 2022	VAN	n/a / varying	COI, n/a / n/a	Absence of clinical failure: (defined as: persistent culture for > = 7 days, recurrence of infection within 30 days of end of COI, 30-day All-cause mortality	n/a (a,c = 0) / n/a	n/a

Abbreviations: ABG = Arterial Blood Gas; CI = Confidence Interval; COI = Continuous Infusion; FEP = Cefepime; h = hour; IA: Intermittent Administration; MEM = Meropenem; min = minutes; PI = Prolonged Infusion; RR = Relative Risk; TZP = Piperacillin & Tazobactam; VAN = Vancomycin

Table S5. Summary of findings table for the outcome "Microbiological eradication".

Study	Antibiotic	Duration / Interval		Results Outcome Microbiological Eradication		
		IA,	PI & COI	Definition of outcome	RR / 95% CI	Significance: (RR = 1 not in 95% CI) / P-value
Beauchamp, 2019	FEP	30 min Infusion / every 8 h	4 h infusion / every 8 h	Defined as having negative follow-up cultures within 72 h of cefepime initiation.	0.97 / 0.88 – 1.08	No / 0.62
Chongcharoenyanon, 2021	TZP	30 min Infusion / every 8 h	4 h infusion / every 8 h	n/a	n/a / n/a	n/a
Shabaan, 2017	MEM	30 min Infusion / every 8 h	4 h infusion / every 8 h	Defined as eradication of organism previously sensitive to meropenem at 7 days of meropenem therapy	1.45 / 1.10 – 1.90	Yes / 0.007
Solorzano, 2019	TZP	30 min Infusion / every 8 h	24 h infusion / n/a	n/a	n/a / n/a	n/a
Zembles, 2021	FEP, MEM, TZP	Infusion 15 - 30 min / varying	FEP & TZP: 4 h infusion / every 8 h; MEM: 3 h infusion / every 8 h;	n/a	n/a / n/a	n/a
Zembles, 2022	FEP, MEM, TZP	< 30 min Infusion / varying	3 - 4 h Infusion / every 8 hours	n/a	n/a/ n/a	n/a
Demirel, 2015	VAN	Infusion 60 min / varying	24 h infusion / n/a	Defined as infants with positive blood cultures at the beginning and became negative at 48 hours	1.10 / 0.61 – 1.98	No / 0.75

Gwee, 2019	VAN	Infusion 60 min / varying	24 h infusion / n/a	Negative blood culture after start (time point n/a)	1.03 / 0.81 – 1.30	No / 0.81
Wysocki, 2022	VAN	n/a / varying	COI, n/a / n/a	Defined as no persistent positive culture for greater or equal than 7 days	n/a / n/a	n/a

Abbreviations: CI = Confidence Interval; COI = Continuous Infusion; FEP = Cefepime; h = hour; IA: Intermittent Administration; MEM = Meropenem; min = minutes; PI = Prolonged Infusion; RR = Relative Risk; TZP = Piperacillin & Tazobactam; VAN = Vancomycin

Supplement I: Forest Plots of Statistically Non-significant Outcomes

Glycopeptides: outcome mortality

Except for the Demirel study, all glycopeptide studies reported no deaths in both study arm. In the Demirel study, there was one death in the COI group (1/35) compared to zero deaths in the IA group (0.5/41) resulting in a relative risk of 2.31 (CI: 0.08 – 66.73).

Outcome clinical success

All beta-lactam studies reported results regarding clinical success. Among patients receiving COI/PI, 77.5% (389/502) experienced clinical success, compared to 76.0% (462/608) for those receiving IA. The pooled RR estimate of 1.02 was statistically non-significant (P = 0.81). The prediction interval included RR = 1. Chi² (P = 0.03) and I² statistic (I² = 59%) indicated substantial heterogeneity, (**Figure S4**).

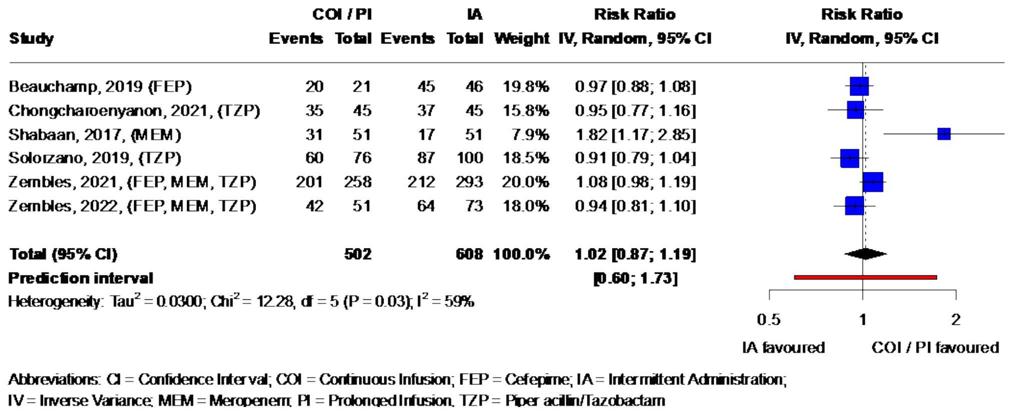

Figure S4. Forest plot assessment of the outcome clinical success for beta-lactams.

For glycopeptides, one study reported results regarding clinical success [9]. Among patients receiving COI treatment, 94.4% (34/36) experienced clinical success compared to 100.0% (41/41) receiving IA. The RR was 0.94 (95% CI = 0.86 – 1.03; P = 0.22), suggesting non-significant differences between intervention and comparison groups.

Outcome microbiological eradication

Two beta-lactam studies reported data regarding microbiological eradication. Among patients receiving PI, 86.1% (62/72) achieved the outcome, compared to 76.3% (74/97) receiving IA. The pooled RR estimate of 1.16 was statistically non-significant (P = 0.44). Chi² (P < 0.01), I² statistics, and visual inspection revealed high heterogeneity among studies (**Figure S5**).

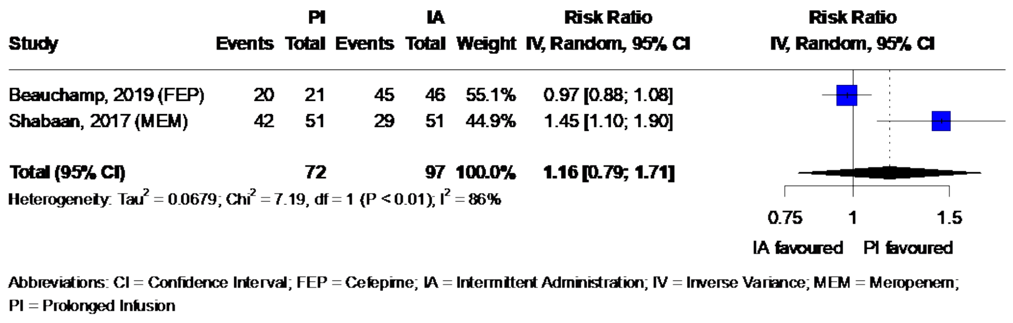

Figure S5. Forest plot assessment of the outcome microbiological eradication for beta-lactams.

One glycopeptide study provided results on microbiological eradication [9]. Among patients receiving COI treatament, 63.6% (7/11) achieved microbiological cure, compared

to 57.9% (11/19) receiving IA. The RR was 1.10 (95% CI = 0.61 – 1.98; p = 0.75), suggesting non-significant differences between intervention and comparison groups.

Outcome target attainment

In a beta-lactam study with 45 participants in each study arm, target attainment data were reported [5]. For 50% ft > MIC with cut-off MICs of 2, 4, 6, 8, 16 and 32 mg/L, the PI group had significantly more patients attaining the target concentration than the IA group (p < 0.01). Equivalent results were found for 50% ft > 4x MIC (p < 0.01) except for a MIC of 32 mg/L (p = 0.17).

Two glycopeptide studies reported data on target attainment. Among patients receiving COI, 78.7% (70/89) attained target concentrations compared to 69.2% (45/65) receiving IA. The pooled RR estimate of 1.19 was statistically non-significant (p = 0.09), and no significant heterogeneity was found in quantitative assessment (**Figure S6**).

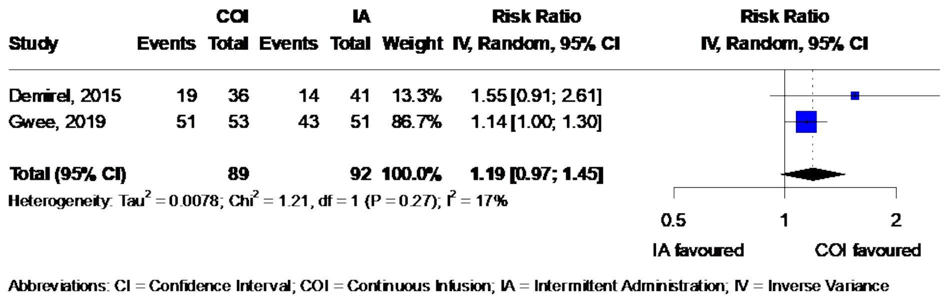

Figure S6. Forest plot assessment of the outcome microbiological eradication for beta-lactams.

Adverse drug reactions (ADRs)

Two studies reported adverse drug reactions (ADRs) of beta-lactams.
Shabaan et al. [3] reported a significantly lower incidence of acute kidney injury of the PI group (5.9%, 3/51) compared to the IA group (23.5%, 12/51; P = 0.02). Non-significant differences in ADRs between study arms are detailed in the extraction table.

All three glycopeptide studies recorded ADRs, and no significant differences between COI and IA were found.

Supplement J: Subgroup Analysis Considerations & Results

Additional considerations on subgroup analyses:

- Variability in half-life, protein binding, and stability exists among different beta-lactams and glycopeptides -> Stratification or subgroup analysis of drugs should be performed.
- Pharmacokinetic (PK) changes occur in different age groups of paediatric patients ->Stratification by age groups is recommended.
- Severity of disease may result in fluid shifts, GFR decline, cardiac output changes, and organ dysfunction. These factors can impact antibiotic concentrations, influencing primary and secondary outcomes. Stratification is advisable to address them as confounding factors.
- Different pathogens exhibit varying MICs (Minimum Inhibitory Concentrations). Stratification by pathogen types, if possible, should be considered.

Subgroup analyses were conducted for cancer, which was the only assessable indication with at least two studies reporting outcomes. (**Figure S7**, **Figure S8**).

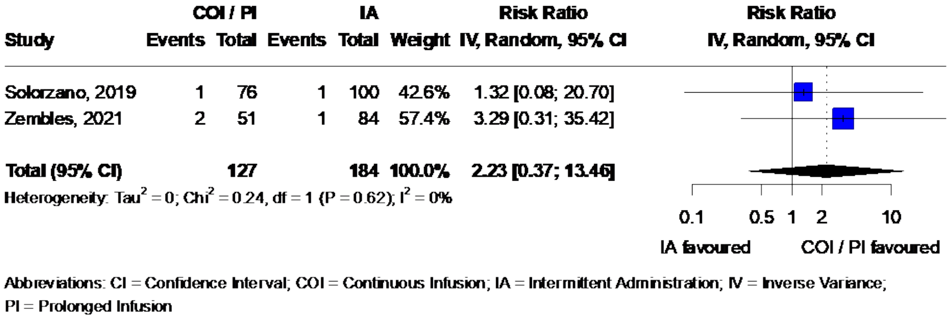

Figure S7. Forest Plot for the Subgroup Cancer, Outcome Mortality.

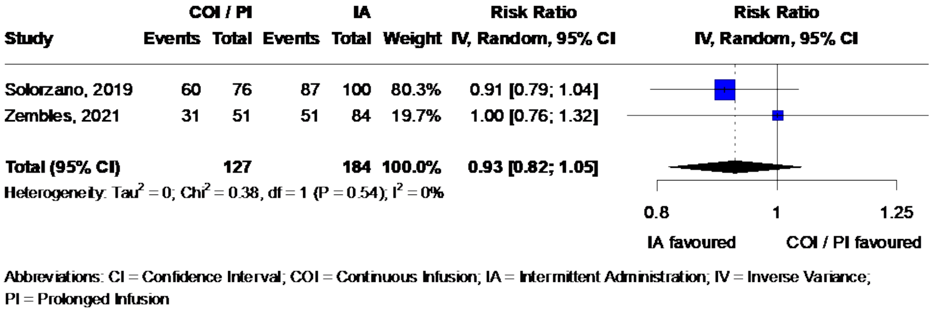

Figure S8. Forest Plot for the Subgroup Cancer, Outcome Clinical Success.

Supplement K: Funnel Plots for Publication Bias Assessment

Funnel plots of primary and secondary outcomes:

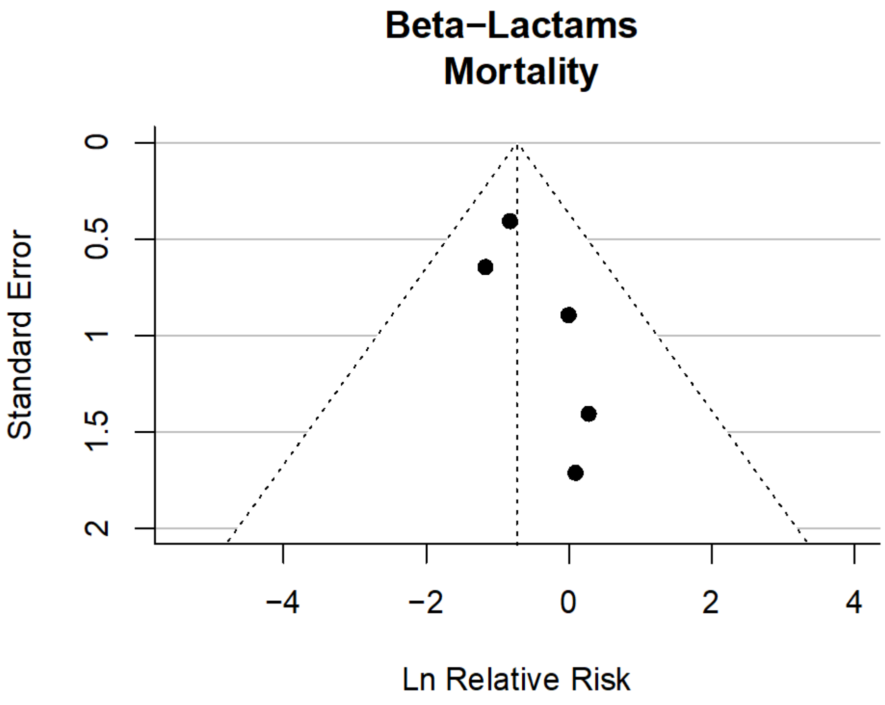

Figure S9. Funnel Plot for the outcome Mortality of Beta-Lactams.

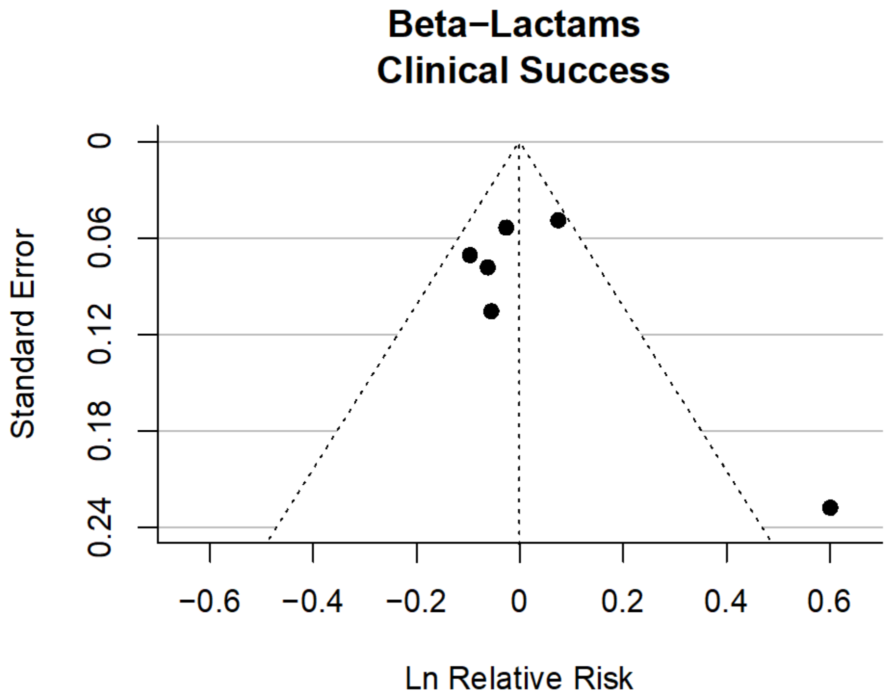

Figure S10. Funnel Plot for the outcome Clinical Success of Beta-Lactams.

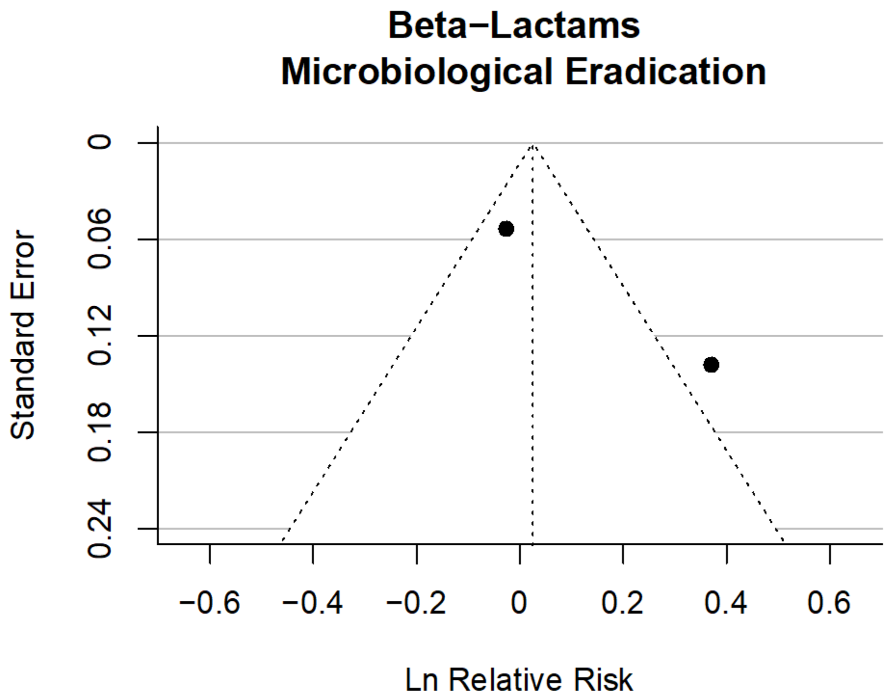

Figure S11. Funnel Plot for the outcome Microbiological Eradication of Beta-Lactams.

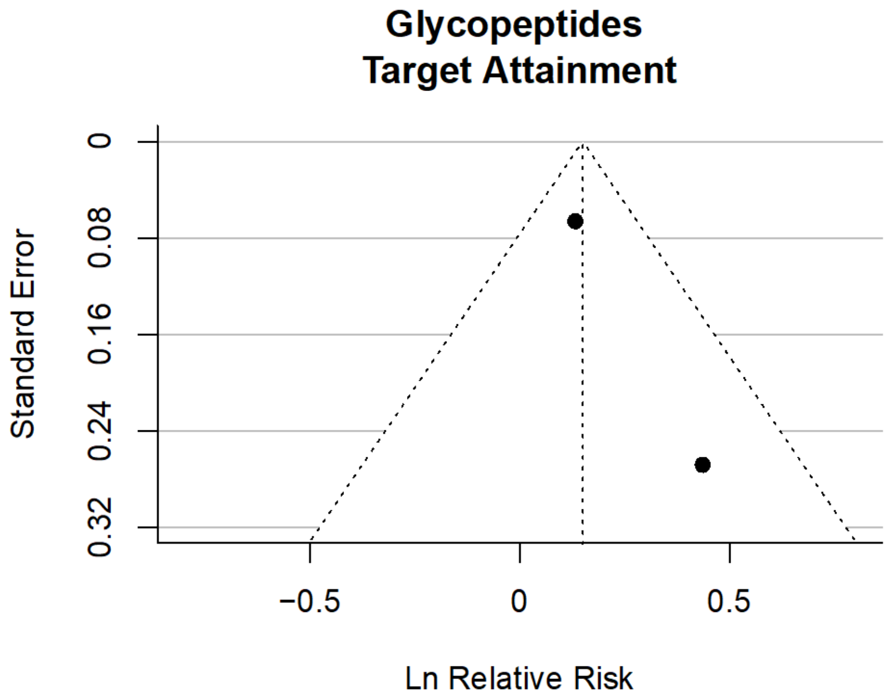

Figure S12. Funnel Plot for the outcome Target Attainment of Glycopeptides.

References

1. Altman, D.G.; Bland, J.M. How to obtain the P value from a confidence interval. *BMJ* **2011**, *343*, d2304-d2304, doi:10.1136/bmj.d2304.
2. Aguinis, H.; Vassar, M.; Wayant, C. On reporting and interpreting statistical significance and p values in medical research. *BMJ Evidence-Based Medicine* **2021**, *26*, 39-42, doi:10.1136/bmjebm-2019-111264.
3. Shabaan, A.E.; Nour, I.; Elsayed Eldegla, H.; Nasef, N.; Shouman, B.; Abdel-Hady, H. Conventional Versus Prolonged Infusion of Meropenem in Neonates With Gram-negative Late-onset Sepsis: A Randomized Controlled Trial. *Pediatr Infect Dis J* **2017**, *36*, 358-363, doi:10.1097/inf.0000000000001445.
4. Solórzano-Santos, F.; Quezada-Herrera, A.; Fuentes-Pacheco, Y.; Rodríguez-Coello, G.; Aguirre-Morales, C.E.; Izelo-Flores, D.; Muñoz-Hernández, O.; Miranda-Novales, M.G.; Labra-Zamora, M.G. Piperacillin/Tazobactam in Continuous Infusion versus Intermittent Infusion in Children with Febrile Neutropenia. *Rev Invest Clin* **2019**, *71*, 283-290, doi:10.24875/ric.19002943.
5. Chongcharoenyanon, T.; Wacharachaisurapol, N.; Anugulruengkitt, S.; Maimongkol, P.; Anunsittichai, O.; Sophonphan, J.; Chatsuwan, T.; Puthanakit, T. Comparison of piperacillin plasma concentrations in a prospective randomised trial of extended infusion versus intermittent bolus of piperacillin/tazobactam in paediatric patients. *International Journal of Infectious Diseases* **2021**, *108*, 102-108, doi:10.1016/j.ijid.2021.05.044.
6. Zembles, T.N.; Schortemeyer, R.; Kuhn, E.M.; Bushee, G.; Thompson, N.E.; Mitchell, M.L. Extended infusion of beta-lactams is associated with improved outcomes in pediatric patients. *Journal of Pediatric Pharmacology and Therapeutics* **2021**, *26*, 187-193, doi:10.5863/1551-6776-26.2.187.
7. Zembles, T.N.; Kuhn, E.M.; Thompson, N.E.; Mitchell, M.L. Extended Infusion b-Lactams for the Treatment of Gram-Negative Bacteremia in Children. *Journal of Pediatric Pharmacology and Therapeutics* **2022**, *27*, 677-681, doi:10.5863/1551-6776-27.7.677.

8. Beauchamp, L.C.; Nichols, K.R.; Knoderer, C.A. Outcomes of Extended Infusion Cefepime in Pediatric Patients. *Infectious Diseases in Clinical Practice* **2019**, *27*, 283-287, doi:10.1097/IPC.0000000000000767.
9. Demirel, B.; Imamoglu, E.; Gursoy, T.; Demirel, U.; Topçuoglu, S.; Karatekin, G.; Ovali, F. Comparison of intermittent versus continuous vancomycin infusion for the treatment of late-onset sepsis in preterm infants. *Journal of Neonatal-Perinatal Medicine* **2015**, *8*, 149-155, doi:10.3233/NPM-15814103.
10. Gwee, A.; Cranswick, N.; McMullan, B.; Perkins, E.; Bolisetty, S.; Gardiner, K.; Daley, A.; Ward, M.; Chiletti, R.; Donath, S.; et al. Continuous versus intermittent vancomycin infusions in infants: A randomized controlled trial. *Pediatrics* **2019**, *143*, doi:10.1542/peds.2018-2179.
11. Wysocki, E.; Tansmore, J. When there is no trough: Continuous infusion vancomycin utilization at a free-standing children's hospital. *Journal of Pediatric Pharmacology and Therapeutics* **2021**, *26*, 525-526, doi:10.5863/1551-6776-26.5.517.

Supplement L: PRISMA Checklist

Section and Topic	Item #	Checklist item	Location where item is reported
**TITLE**			
Title	1	Identify the report as a systematic review.	P. 1 line 3
**ABSTRACT**			
Abstract	2	See the PRISMA 2020 for Abstracts checklist.	P. 1 line 13-27
**INTRODUCTION**			
Rationale	3	Describe the rationale for the review in the context of existing knowledge.	p. 1-3<br>line 30-100
Objectives	4	Provide an explicit statement of the objective(s) or question(s) the review addresses.	p. 3 line 101-105
**METHODS**			
Eligibility criteria	5	Specify the inclusion and exclusion criteria for the review and how studies were grouped for the syntheses.	p. 11-12 line 247-249; Table 7
Information sources	6	Specify all databases, registers, websites, organisations, reference lists and other sources searched or consulted to identify studies. Specify the date when each source was last searched or consulted.	p. 13 line 252-263
Search strategy	7	Present the full search strategies for all databases, registers and websites, including any filters and limits used.	p. 13 line 252-263; Supplementary material A, B and C
Selection process	8	Specify the methods used to decide whether a study met the inclusion criteria of the review, including how many reviewers screened each record and each report retrieved, whether they worked independently, and if applicable, details of automation tools used in the process.	p. 13 line 266-269
Data collection	9	Specify the methods used to collect data from reports, including how many reviewers collected data from each report, whether they	p. 13 line 272-282

Section and Topic	Item #	Checklist item	Location where item is reported
process		worked independently, any processes for obtaining or confirming data from study investigators, and if applicable, details of automation tools used in the process.	
Data items	10a	List and define all outcomes for which data were sought. Specify whether all results that were compatible with each outcome domain in each study were sought (e.g. for all measures, time points, analyses), and if not, the methods used to decide which results to collect.	p. 12 Table 7; Supplement D
	10b	List and define all other variables for which data were sought (e.g. participant and intervention characteristics, funding sources). Describe any assumptions made about any missing or unclear information.	p. 13 line 275-282; Supplement H
Study risk of bias assessment	11	Specify the methods used to assess risk of bias in the included studies, including details of the tool(s) used, how many reviewers assessed each study and whether they worked independently, and if applicable, details of automation tools used in the process.	p. 13 line 285-290
Effect measures	12	Specify for each outcome the effect measure(s) (e.g. risk ratio, mean difference) used in the synthesis or presentation of results.	p. 13 line 276-277; Supplement D
Synthesis methods	13a	Describe the processes used to decide which studies were eligible for each synthesis (e.g. tabulating the study intervention characteristics and comparing against the planned groups for each synthesis (item #5)).	p. 13 line 293-298
	13b	Describe any methods required to prepare the data for presentation or synthesis, such as handling of missing summary statistics, or data conversions.	p. 13 line 293-298
	13c	Describe any methods used to tabulate or visually display results of individual studies and syntheses.	p. 13 line 295-298
	13d	Describe any methods used to synthesize results and provide a rationale for the choice(s). If meta-analysis was performed, describe the model(s), method(s) to identify the presence and extent of statistical heterogeneity, and software package(s) used.	p. 13 line 293-298; p. 14 line 300-307
	13e	Describe any methods used to explore possible causes of heterogeneity among study results (e.g. subgroup analysis, meta-regression).	p. 14 line 295-303
	13f	Describe any sensitivity analyses conducted to assess robustness of the synthesized results.	p. 13 line 287-289
Reporting bias assessment	14	Describe any methods used to assess risk of bias due to missing results in a synthesis (arising from reporting biases).	p. 14 line 310-314

Section and Topic	Item #	Checklist item	Location where item is reported
Certainty assessment	15	Describe any methods used to assess certainty (or confidence) in the body of evidence for an outcome.	p. 14 line 317-319
**RESULTS**			
Study selection	16a	Describe the results of the search and selection process, from the number of records identified in the search to the number of studies included in the review, ideally using a flow diagram.	p. 3-4 line 108-117
	16b	Cite studies that might appear to meet the inclusion criteria, but which were excluded, and explain why they were excluded.	Supplement E
Study characteristics	17	Cite each included study and present its characteristics.	p. 6-7 line 130-132 (Table 4 and 5)
Risk of bias in studies	18	Present assessments of risk of bias for each included study.	p. 5 line 119-128; Supplement F
Results of individual studies	19	For all outcomes, present, for each study: (a) summary statistics for each group (where appropriate) and (b) an effect estimate and its precision (e.g. confidence/credible interval), ideally using structured tables or plots.	p. 7 line 138-149; p. 9 Table 6; Supplement I and J
Results of syntheses	20a	For each synthesis, briefly summarise the characteristics and risk of bias among contributing studies.	p. 9 Table 6 and Supplement F
	20b	Present results of all statistical syntheses conducted. If meta-analysis was done, present for each the summary estimate and its precision (e.g. confidence/credible interval) and measures of statistical heterogeneity. If comparing groups, describe the direction of the effect.	p. 5 line 138-149; Supplement I and J
	20c	Present results of all investigations of possible causes of heterogeneity among study results.	p. 8 line 141-143
	20d	Present results of all sensitivity analyses conducted to assess the robustness of the synthesized results.	p. 5 line 126-128; Supplement G
Reporting biases	21	Present assessments of risk of bias due to missing results (arising from reporting biases) for each synthesis assessed.	p. 8 line 156-158;

Section and Topic	Item #	Checklist item	Location where item is reported
			Supplement K
Certainty of evidence	22	Present assessments of certainty (or confidence) in the body of evidence for each outcome assessed.	p. 8 line 161-162
**DISCUSSION**			
Discussion	23a	Provide a general interpretation of the results in the context of other evidence.	p. 10 line 167-179
	23b	Discuss any limitations of the evidence included in the review.	p. 11 line 211-225
	23c	Discuss any limitations of the review processes used.	p. 11 line 226-232
	23d	Discuss implications of the results for practice, policy, and future research.	p. 10 line 197-206 and p. 11 line 235-241
**OTHER INFORMATION**			
Registration and protocol	24a	Provide registration information for the review, including register name and registration number, or state that the review was not registered.	p. 11 line 246
	24b	Indicate where the review protocol can be accessed, or state that a protocol was not prepared.	p. 11 line 246
	24c	Describe and explain any amendments to information provided at registration or in the protocol.	p. 11 line 247; Supplement A
Support	25	Describe sources of financial or non-financial support for the review, and the role of the funders or sponsors in the review.	p. 15 line 341
Competing interests	26	Declare any competing interests of review authors.	p. 15 line 343
Availability of data, code and	27	Report which of the following are publicly available and where they can be found: template data collection forms; data extracted from included studies; data used for all analyses; analytic code; any other materials used in the review.	p. 15 line 345

**Section and Topic**	**Item #**	**Checklist item**	**Location where item is reported**
other materials			

From: Page MJ, McKenzie JE, Bossuyt PM, Boutron I, Hoffmann TC, Mulrow CD, et al. The PRISMA 2020 statement: an updated guideline for reporting systematic reviews. BMJ 2021;372:n71. doi: 10.1136/bmj.n71.
